# A new tool for tuberculosis vaccine screening: *Ex vivo* Mycobacterial Growth Inhibition Assay indicates BCG-mediated protection in a murine model of tuberculosis

**DOI:** 10.1186/s12879-016-1751-4

**Published:** 2016-08-12

**Authors:** Andrea Zelmer, Rachel Tanner, Elena Stylianou, Timon Damelang, Sheldon Morris, Angelo Izzo, Ann Williams, Sally Sharpe, Ilaria Pepponi, Barry Walker, David A. Hokey, Helen McShane, Michael Brennan, Helen Fletcher

**Affiliations:** 1 London School of Hygiene and Tropical Medicine, London, UK; 2 The Jenner Institute, Oxford University, Oxford, UK; 3 Center for Biologics Evaluation and Research, Silver Spring, MD USA; 4 Colorado State University, Fort Collins, CO USA; 5 Public Health England, Porton Down, UK; 6 Animal and Plant Health Agency, Weybridge, UK; 7 Aeras, Rockville, MD USA; 8 Translational Research Unit, National Institute for Infectious Diseasess “Lazzaro Spallanzani”, Rome, Italy

**Keywords:** Mycobacteria, Tuberculosis, Vaccines, Growth inhibition assay

## Abstract

**Background:**

In the absence of a validated animal model and/or an immune correlate which predict vaccine-mediated protection, large-scale clinical trials are currently the only option to prove efficacy of new tuberculosis candidate vaccines. Tools to facilitate testing of new tuberculosis (TB) vaccines are therefore urgently needed.

**Methods:**

We present here an optimized *ex vivo* mycobacterial growth inhibition assay (MGIA) using a murine *Mycobacterium tuberculosis* infection model. This assay assesses the combined ability of host immune cells to inhibit mycobacterial growth in response to vaccination. C57BL/6 mice were immunized with Bacillus Calmette-Guérin (BCG) and growth inhibition of mycobacteria by splenocytes was assessed. Mice were also challenged with *Mycobacterium tuberculosis* Erdman, and bacterial burden was assessed in lungs and spleen.

**Results:**

Using the growth inhibition assay, we find a reduction in BCG CFU of 0.3–0.8 log10 after co-culture with murine splenocytes from BCG vaccinated versus naïve C57BL/6 mice. BCG vaccination in our hands led to a reduction in bacterial burden after challenge with *Mycobacterium tuberculosis* of approx. 0.7 log10 CFU in lung and approx. 1 log10 CFU in spleen. This effect was also seen when using *Mycobacterium smegmatis* as the target of growth inhibition. An increase in mycobacterial numbers was found when splenocytes from interferon gamma-deficient mice were used, compared to wild type controls, indicating that immune mechanisms may also be investigated using this assay.

**Conclusions:**

We believe that the *ex vivo* mycobacterial growth inhibition assay could be a useful tool to help assess vaccine efficacy in future, alongside other established methods. It could also be a valuable tool for determination of underlying immune mechanisms.

**Electronic supplementary material:**

The online version of this article (doi:10.1186/s12879-016-1751-4) contains supplementary material, which is available to authorized users.

## Background

Tuberculosis (TB) is a world-wide public health problem and the biggest cause of death due to a single pathogen. It is estimated that in 2014, 9.6 million people developed the disease, and 1.5 million died from it [1]. Bacillus Calmette-Guérin (BCG) is the only available vaccine. It confers reliable protection against severe TB in infants and children, but its efficacy in adults is extremely variable (0–80 %), and a new vaccine is urgently needed to control the spread of the infection [2, 3]. Progress towards this goal has been slow. There are several reasons for this stagnation. *Mycobacterium tuberculosis* (*Mtb*) can persist in infected persons for years without causing disease or symptoms. Vaccine-mediated protection is therefore difficult to measure, resulting in a need for lengthy and costly clinical trials to establish vaccine efficacy. Additionally, only a handful of new vaccine candidates are currently under development, and funders have become more reluctant to provide large investments due to a risk of vaccine failure.

There is currently no validated immune correlate that predicts efficacy (such as antibody titres used for other infections) and this severely hampers the development and pre-clinical and clinical testing of TB vaccine candidates. Additionally, as highlighted by McShane and Williams [4] we do not have a single validated animal model for the screening of TB vaccine candidates, although head-to-head testing of TB vaccine candidates across a range of animal models can be reasonably used to demonstrate that vaccines are efficacious. Further, Henao-Tamayo et al. [5] argue that TB vaccines should be tested against different clinical isolates of *Mtb*, since the BCG Pasteur vaccine used in their study displayed varying levels of protection against different *Mtb* isolates in mouse and guinea pig models. A novel tool that would allow vaccine screening in different species may be more time- and cost-efficient than in vivo challenge experiments and could therefore help to accelerate vaccine development. The ability to test several different isolates or lineages of *Mtb* using cells from the same animal would reduce the number of animals needed, and the cost involved in these experiments.

Here, we present an optimized *ex vivo* mycobacterial growth inhibition assay (MGIA) for assessment of the summative vaccine-mediated host capacity to control mycobacterial growth. Several variations of mycobacterial growth inhibition assays have been described previously [6–9]. The assay described here involves direct co-culture of mouse splenocytes with mycobacteria, and subsequent measurement of mycobacterial growth inhibition. This particular variation of the assay has previously been shown to distinguish between naïve and immunized individuals in mice as well as humans by use of PBMC; however, the differences in mycobacterial burden between those two groups were small [10, 11]. To allow detection of a range of efficacies from different vaccine candidates, we aimed to achieve a difference of >0.5 log10 colony forming units (CFU) between BCG-naïve and BCG-immunized groups in our mouse model of TB. Our optimized assay produces a difference of up to 0.8 log10 CFU in our hands. Growth inhibition was also observed when using fast-growing *Mycobacterium smegmatis* (*Msm*) as the target bacteria in the MGIA. An in vivo challenge with the *Mtb* Erdman laboratory strain resulted in a ~0.7 log10 reduction in mycobacterial burden in the lungs and a 1 log10 reduction in the spleens of immunized animals, compared to unimmunized controls. We further demonstrate that an MGIA can show the importance of non-vaccine mediated interferon gamma (IFNγ)-dependent activity by using IFNγ-deficient (IFNγ^-/-^) mice. Collectively, our data suggest that MGIAs could be a promising tool for screening vaccine candidates pre-clinically, as well as determine the underlying immune mechanisms.

## Methods

### Animals

For immunization experiments, female C57BL/6 mice were acquired from Charles River UK at 5–7 weeks of age. Animals were acclimatized for at least 5 days before the start of any experimental procedure. Female B6.129S7-*Ifng*^*tm1Ts*^/J (IFNγ^-/-^) and C57BL/6 wild type (WT) controls bred in-house were used at 10–12 weeks of age. Group sizes of 5–6 mice were used as indicated throughout the manuscript.

### Mycobacteria and culture conditions

BCG SSI and BCG Pasteur Aeras strains were obtained from Aeras (Rockville, MD, USA) as frozen aliquots. These were stored at -80 °C until needed. *M. smegmatis* was grown in 7H9 media with 10 % OADC, 0.5 % Glycerol and 0.05 % Tween80. At late log phase, bacteria were washed once with phosphate-buffered saline (PBS) + 0.05 % Tween80, and resuspended in PBS + 10 % Glycerol. Aliquots were frozen and stored at -80 °C until needed.

### Immunization

Bacteria were thawed at room temperature and diluted to a final concentration of 2–5 × 10^6^ CFU/ml in physiological saline solution for irrigation (Baxter Healthcare, Newbury, UK). Each animal received a subcutaneous injection of 100 μl BCG (immunized groups) or physiological saline solution (control groups) into the left or right leg flap. Animals were rested for 6 weeks (unless indicated otherwise) before an *ex vivo* mycobacterial growth inhibition assay or infection with *M. tuberculosis* was carried out.

### *Ex vivo* Mycobacterial Growth Inhibition Assay (MGIA)

Six weeks after immunization (unless specified otherwise), spleens were removed aseptically and single cell suspensions of splenocytes isolated by mechanical disruption of spleens through a 100 μm cell strainer. After lysis of red blood cells, single cell suspensions containing the desired number of total splenocytes per 300 μl were made up in antibiotic-free media (RPMI-1640 (Sigma-Aldrich, Dorset, UK) + 10 % heat-inactivated FBS (Labtech International Ltd, Uckfield, UK) + 2 mM L-Glutamine (Fisher Scientific, Loughborough, UK), and 300 μl aliquots were added to 2 ml screw cap tubes (Sarstedt, Nümbrecht, Germany). Mycobacteria were diluted in sufficient volume for all samples in the same media to a concentration of 90 to 3800 CFU per 300 μl as indicated for individual experiments. 300 μl aliquots of bacteria were added to the splenocytes, and the splenocyte-mycobacteria co-culture was then incubated on 360° tube rotators (VWR International, Lutterworth, UK) at 37 °C for 4 days.

After 4 days of incubation, the 2 ml screw cap tubes were centrifuged at 12,000 rpm in a bench-top microcentrifuge. The supernatants were removed (except 100 μl), and 400 μl of water was added to each tube to lyse host cells. The tubes were then vortexed briefly three times, with an incubation at room temperature for 5 min between each of the vortex steps. Each cell lysate (approx. 500 μl total) was then added to a MGIT tube (BD, Oxford, UK) and incubated in a BACTEC MGIT liquid culture system (BD) until registered positive.

To convert time to positivity (TTP) to bacterial numbers (CFU), a standard curve was used. To produce the standard curve, 500 μl of 10-fold dilutions of the mycobacterial strains were inoculated into the MGIT tubes, and TTP was plotted against CFU obtained from plating aliquots of the mycobacteria on 7H11 agar plates containing 10 % OADC supplement (Yorlab, York, UK) and 0.5 % glycerol. A linear regression analysis was carried out using GraphPad Prism version 6, and the resulting equation was used to convert TTP to CFU. Data are presented here as total number of CFUs per sample, as determined by use of a standard curve (Additional file 1: Figure S1). The difference between the medians of respective groups is described in the text and figures as Δ X log, and was calculated by subtracting the median of the test group (immunized or IFNγ-deficient mice) from the median of the control group (unimmunized or wild type mice).

### *Infection of mice with* M. tuberculosis

Female C57BL/6 mice were infected intranasally with *M. tuberculosis* Erdman (BEI Resources, Manassas, VA, USA) 6 weeks after immunization and kept in isolators under BSL-3 containment. Frozen aliquots as received from BEI Resources were thawed at room temperature, and diluted in saline to a concentration of 1.4x10^4^ CFU/ml. Mice were anaesthetized by an intraperitoneal injection of a combination of Ketamine (50 mg/kg; Ketalar, Pfizer Itd, Kent, UK) and Xylazine (10 mg/kg; Rompun; Berkshire, UK) in saline. Each animal then received 50 μl of the inoculum, estimated to contain 700 CFU. The number of bacteria in the inoculum was confirmed by plating aliquots on 7H11 agar plates containing 10 % OADC and 0.5 % glycerol.

Four weeks after infection, animals were killed by cervical dislocation. Lungs and spleens were removed aseptically and homogenized by mechanical disruption in sterile PBS. A series of 10-fold dilutions of tissue homogenates in PBS with 0.05 % Tween 80 were plated onto 7H11 agar plates with 10 % OADC supplement and 0.5 % glycerol. Plates were incubated at 37 °C and colonies counted after 3 weeks.

### Statistical analysis

Statistical analysis was carried out using GraphPad Prism software Version 6 (GraphPad, La Jolla, CA, USA). The specific test used is indicated in each figure legend.

## Results

### *Comparison of BCG SSI and BCG Pasteur in an* ex vivo *MGIA*

**Fig. 1:**
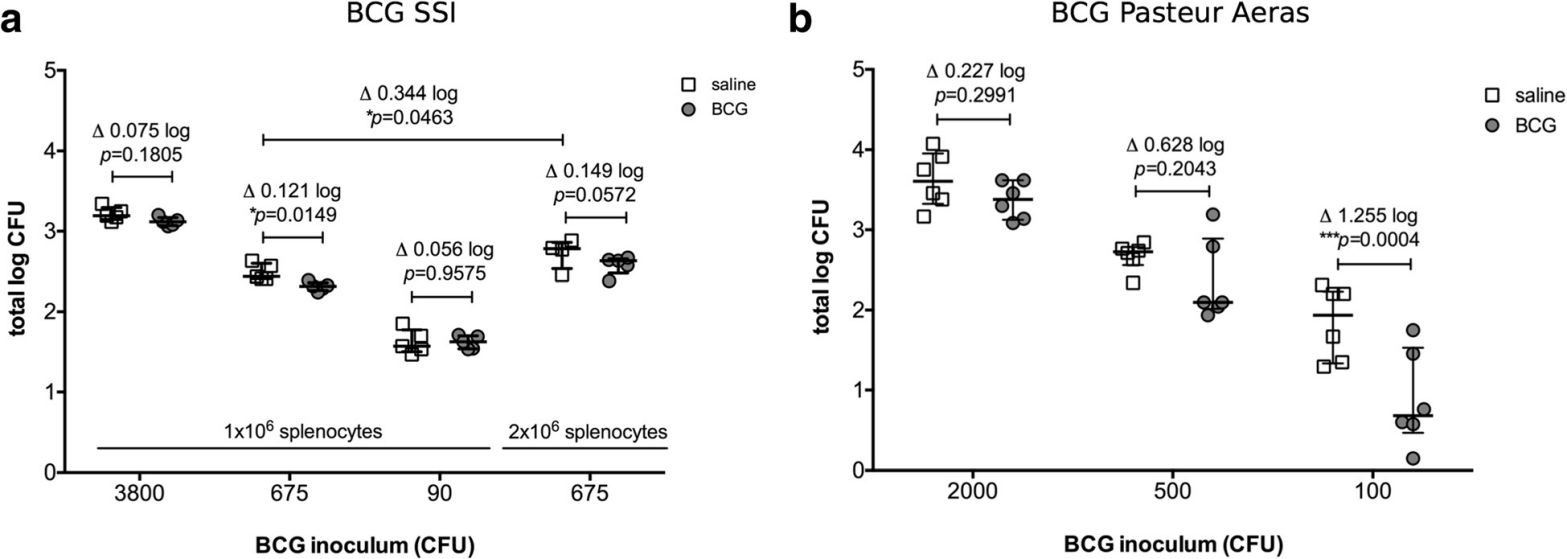
*Ex vivo* MGIA comparing growth inhibition conferred by BCG SSI and BCG Pasteur Aeras. **a** 1 × 10^6^ splenocytes or 2 × 10^6^ splenocytes from mice immunized with BCG SSI (grey circles) or given saline (open squares) were co-cultured with 3800, 675, or 90 CFU of BCG SSI. **b** 1 × 10^6^ splenocytes from mice immunized with BCG Pasteur Aeras (grey circles) or given saline (open squares) were co-cultured with 2000, 500, or 100 CFU of BCG Pasteur Aeras. Splenocytes were obtained from a total of 5 immunized and 5 control animals (**a**) or a total of 6 immunized and 6 control animals (**b**). Aliquots from each spleen were cultured with different numbers of mycobacteria as indicated, and are represented by individual data points. Error bars represent the median +/- interquartile range. Statistical significance was tested using the unpaired t test function in GraphPad Prism

As a first step to maximize the difference in bacterial burden between BCG immunized and control groups in the MGIA, we investigated growth inhibition using two different BCG strains, BCG SSI and BCG Pasteur Aeras, an early passage strain of BCG Pasteur. Groups of 5 and 6 CB57BL/6 mice were immunized with BCG SSI and BCG Pasteur Aeras, respectively, or given saline as a control, and an *ex vivo* MGIA was carried out to assess mycobacterial growth inhibition of the same bacterial strains by host splenocytes at 4 or 6 weeks after immunization (Fig. 1). 1 × 10^6^ cells and three different inocula of BCG were used for the MGIA as indicated in Fig. 1. The strongest growth inhibition of BCG SSI was found using 675 CFU. This led to a reduction of 0.121 log10 CFU in the immunized group as compared to the control group. No significant differences were found using 3800 CFU or 90 CFU (Fig. 1a). In contrast, using 100 CFU BCG Pasteur Aeras lead to a >1 log10 CFU reduction in bacterial numbers in the immunized group (Fig. 1b). Growth inhibition was also observed with higher inocula of BCG Pasteur Aeras, and although these were not statistically significant, the differences between immunized and control groups increased with lower inocula of BCG. Notably, significantly higher overall bacterial burdens were observed when a higher number of splenocytes (2 × 10^6^) was used (Fig. 1a; 675 CFU, 1 × 10^6^ vs 2 × 10^6^ splenocytes). This was expected as BCG grows intracellularly in macrophages, and a higher overall number of splenocytes would provide more host cells for mycobacteria to grow in; however, we also observed a trend towards a greater effect on growth inhibition under these conditions (a reduction of 0.149 log10 CFU vs 0.121 log10 CFU).

### *The number of target bacteria and the number of host cells both influence MGIA outcome*

**Fig. 2:**
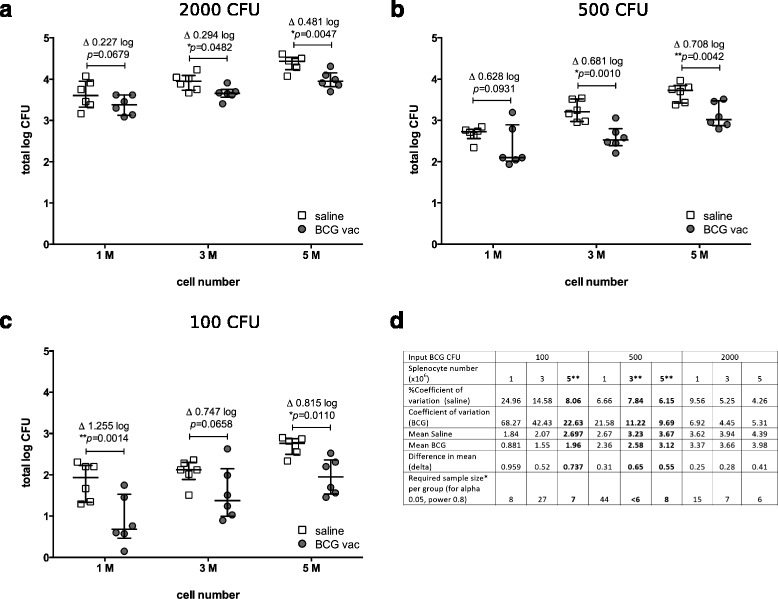
Optimisation of the *ex vivo* MGIA using BCG Pasteur Aeras. **a** 1 × 10^6^, 3 × 10^6^, and 5 × 10^6^ splenocytes (1 M, 3 M, and 5 M, respectively) from mice immunized with BCG Pasteur Aeras (grey circles) or given saline (open squares) were co-cultured with 2000 CFU (**a**), 500 CFU (**b**) or 100 CFU (**c**) of BCG Pasteur Aeras. Splenocytes were obtained from a total of 6 immunized and 6 control animals. Aliquots from each spleen were cultured with different numbers of mycobacteria as indicated, and are represented by individual data points. Error bars represent the median +/- interquartile range. Statistical significance was tested using the unpaired t test function in GraphPad Prism. **d** Analysis of variation of the data shown in **a**-**c**. *Sample sizes (alpha 0.05, power 0.8) were calculated using the mean values of BCG immunized and naïve groups and using the standard deviation of the BCG group (standard deviation was greater in the BCG groups than in the saline groups). **Optimal conditions for vaccine candidate testing determined as delta >0.5 log CFU, CoV < 50 % and required sample size < 10 per group. The analysis was carried out using STATA software

Based on these observations and in order to further improve assay conditions, we proceeded to titrate both the BCG Pasteur Aeras input inoculum (2000, 500, or 100 CFU) and the number of splenocytes used (1, 3, or 5 × 10^6^; Fig. 2). Data shown in Fig. 1b are included for comparison. Significant growth inhibition in the immunized versus the control group was observed under several conditions, ranging from ~0.3 log10 to ~1.2 log10 difference between medians. Again, we observed more bacterial growth overall if more splenocytes were present, and we found that a lower BCG input inoculum and a higher number of splenocytes generally increased the growth inhibition effect in immunized groups. However, lower bacterial or splenocyte numbers also increased the variability within groups (measured as % Coefficient of Variation; Fig. 2d). We determined minimal conditions for vaccine candidate testing as delta >0.5 log CFU, CoV < 50 % and required sample size < 10 per group. These parameters were met by three of the conditions: 5 × 10^6^ cells + 100 CFU; 5 × 10^6^ cells + 500 CFU; and 3 × 10^6^ cells + 500 CFU (indicated in bold script in Fig. 2d). Taking into account that the effect size was greatest using 5 × 10^6^ splenocytes + 100 CFU, we determined this as the optimal condition using BCG Pasteur Aeras and C57BL/6 mice. Variability and reproducibility between experiments is a further important consideration. We have repeated the MGIA under these conditions on four independent occasions (Additional file 2: Figure S2a) and were able to detect significant differences ranging from 0.3–0.8 log10 CFU between immunized and unimmunized groups in three of those experiments. Pooling of data points from all experiments leads to a highly significant difference of 0.48 log10 CFU between immunized and control groups (Additional file 2: Figure S2b).

**Fig. 3:**
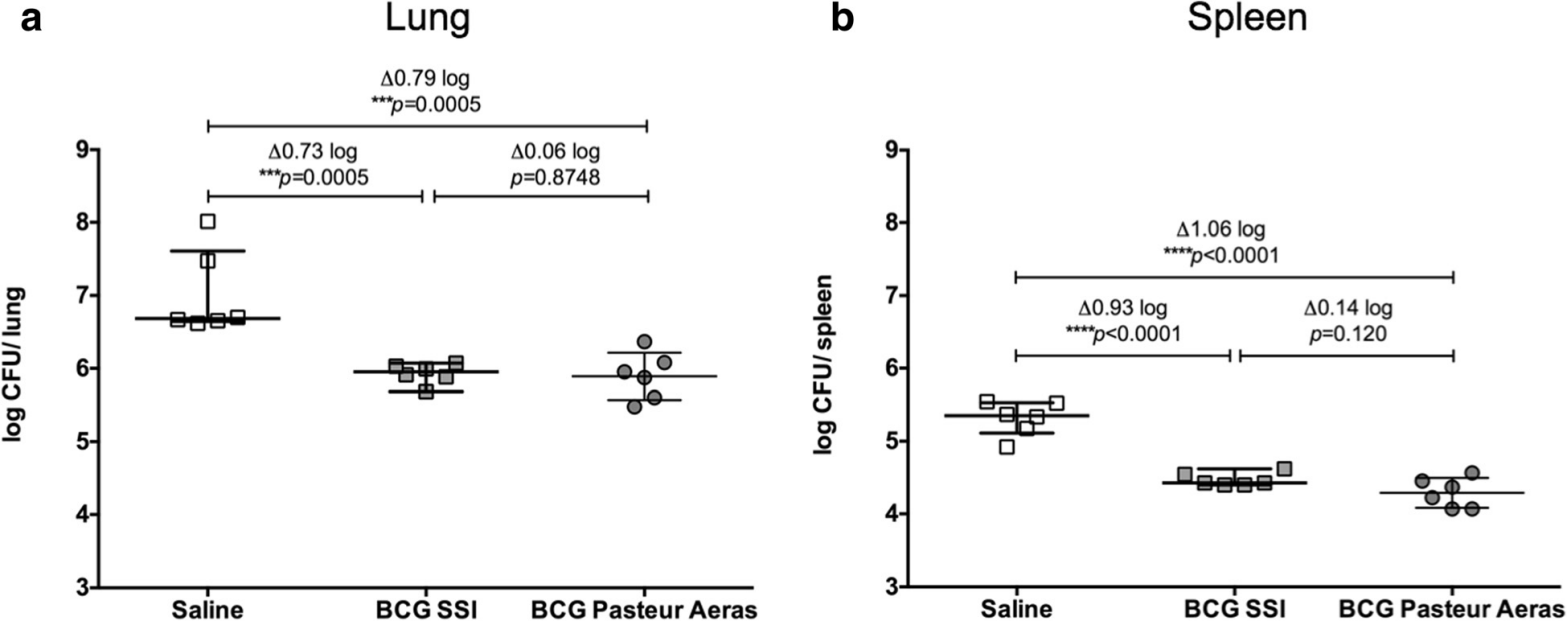
Protection conferred to infection with *M. tuberculosis* by BCG SSI and BCG Pasteur Aeras. Groups of 6 C57BL/6 mice were infected 6 weeks after immunization with BCG SSI (grey squares) or BCG Pateur Aeras (grey circles) via the intranasal route with 700 CFU *M. tuberculosis* Erdman. Control animals received saline at the time of immunization (open squares). CFUs per organ were determined 4 weeks after infection in lungs (**a**) and spleens (**b**). Each data point represents an individual animal. Error bars represent the median +/- interquartile range. Statistical significance was determined by one-way ANOVA with Holm-Sidak correction for multiple comparisons using GraphPad Prism

To assess how our *ex vivo* assay compares to an in vivo infection with virulent *M. tuberculosis*, groups of 6 C57BL/6 mice were immunized with BCG SSI, BCG Pasteur Aeras, or saline (control group), rested for 6 weeks, and then challenged with 700 CFU *Mtb* Erdman via the intranasal route. Bacterial burden in lungs (Fig. 3a) and spleens (Fig. 3b) was established 4 weeks after infection. We found that protection was conferred by both BCG strains, with a significant reduction of bacterial burden in the lung by 0.73 log10 CFU (BCG SSI) or 0.79 log10 CFU (BCG Pasteur Aeras), and in the spleen by 0.93 log10 CFU (BCG SSI) or 1.06 log10 CFU (BCG Pasteur Aeras), compared to control animals. There was no significant difference in bacterial burden in either lung or spleen between the two groups immunized with the different BCG strains.

### *BCG-immunized and control groups can be distinguished in an MGIA by using fast-growing* Mycobacterium smegmatis

**Fig. 4:**
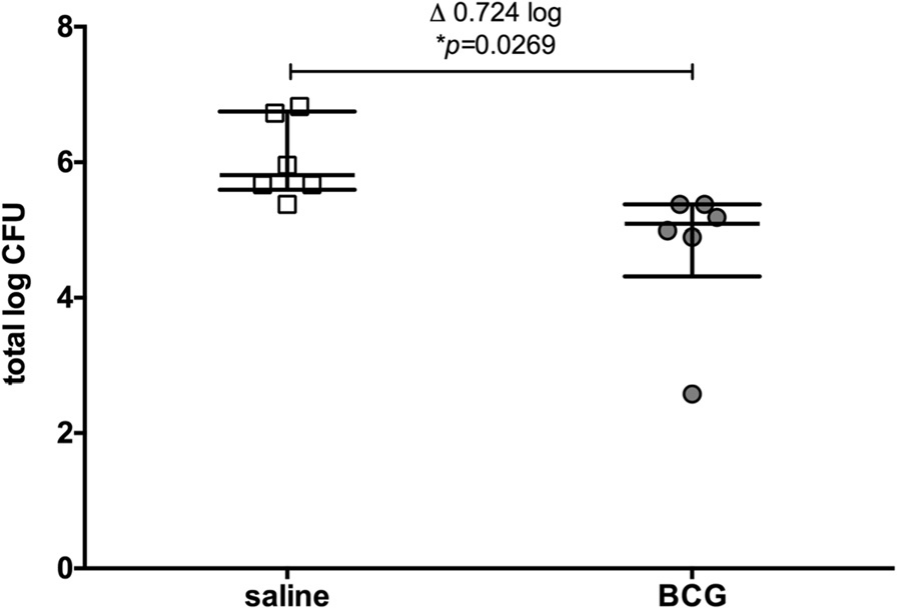
*Ex vivo* MGIA using *M. smegmatis* distinguishes between immunized and control animals. 5 × 10^6^ splenocytes from mice immunized with BCG Pasteur Aeras (grey circles) or given saline (open squares) were co-cultured with 50 CFU of *M. smegmatis* 5 weeks after immunization. Splenocytes were obtained from 6 immunized and 6 control animals, each represented by an individual data point. Error bars represent the median +/- interquartile range. Statistical significance was tested using the unpaired t test function in GraphPad Prism

In an effort to further optimize the MGIA with regards to experiment time, the window between immunized and control groups, and ease of handling, we used the fast growing, non-pathogenic *Mycobacterium smegmatis* as the target bacteria for growth inhibition (Fig. 4). A difference of 0.7 log10 CFU was achieved between the two experimental groups (splenocytes from control mice or from BCG Pasteur Aeras immunized mice), which is equivalent to using BCG Pasteur Aeras. The overall bacterial burden was higher compared to BCG; however, based on the faster doubling time of *Msm*, this was expected. The overall experiment time was reduced by approx. 7 days.

### *MGIA reflects non-vaccine mediated, interferon gamma-dependent responses that are important for control of mycobacterial growth*

**Fig. 5:**
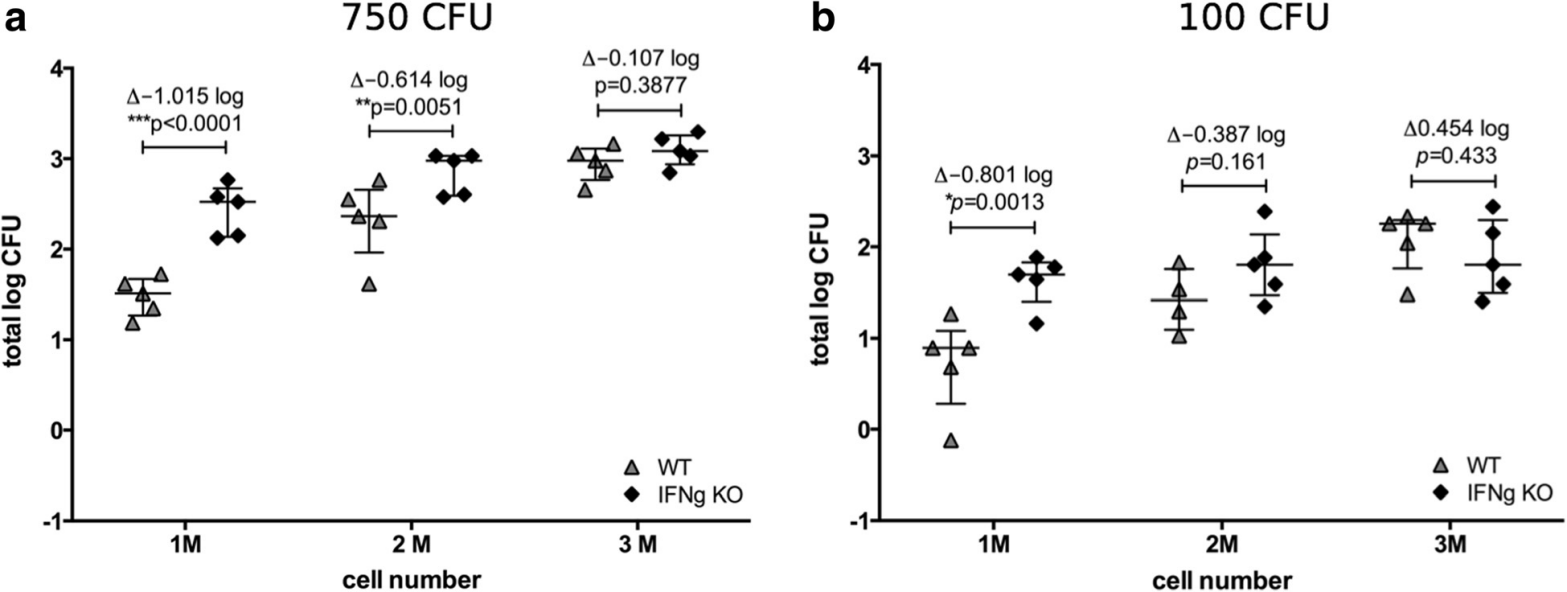
*Ex vivo* MGIA reflects importance of IFNγ for growth control of mycobacteria. 1 × 10^6^, 2 × 10^6^, and 3 × 10^6^ splenocytes from IFNγ-deficient (filled diamonds) or wild type mice (grey triangles) were co-cultured with 750 CFU (**a**) or 100 CFU (**b**) of BCG Pasteur Aeras. Splenocytes were obtained from a total of 5 IFNγ-deficient and 5 wild type animals. Aliquots from each spleen were cultured with different numbers of mycobacteria as indicated, and are represented by individual data points. Error bars represent the median +/- interquartile range. Statistical significance was tested using the unpaired t test function in GraphPad Prism

To characterize the assay and its potential for use in studies of immune mechanisms, we used splenocytes from interferon-gamma (IFNγ)-deficient C57BL/6 mice (IFNγ^-/-^) and assessed their ability to control mycobacterial growth *ex vivo* in comparison to splenocytes from wild type C57BL/6 mice. Here, the number of splenocytes (1, 2, or 3 × 10^6^ cells) was titrated against two different BCG Pasteur Aeras inocula (750 CFU and 100 CFU; Fig. 5). The largest detectable difference was a 1 log10 increase in CFU in the IFNγ^-/-^ splenocytes when using 1 × 10^6^ cells and 750 CFU BCG; using 100 CFU, a 0.8 log10 difference in CFU was observed. This is in concordance with the crucial role of IFNγ in the control of mycobacterial growth in vivo demonstrated widely in the literature [12–14]. Interestingly, the dynamics in this case are different from the vaccine-induced growth control described above (Figs. 1, 2, 3 and 4). An increase in the number of splenocytes led to a smaller difference between the two groups, whilst the opposite trend was observed when comparing splenocytes from immunized and unimmunized mice. Similarly, the higher bacterial inoculum led to overall larger differences between the groups, whilst the opposite was the case in the experiments above.

## Discussion

The first step towards optimization of an *ex vivo* mycobacterial growth inhibition assay for pre-clinical vaccine testing was to compare two different BCG strains, BCG SSI and BCG Pasteur Aeras (Fig. 1). We found that using BCG Pasteur Aeras as both the strain for immunization and the strain for assessment of *ex vivo* growth inhibition led to larger differences between immunized and control groups (Fig. 1b), compared to when BCG SSI was used as the MGIA inoculum and vaccine (Fig. 1a). Importantly, this assay assesses growth inhibition *ex vivo*, and thus provides a snapshot of the ability of the immune system to control mycobacterial growth at one specific time point. The effect of BCG sub-strains on the elicited host immune response and conferred protection has been investigated previously. Irwin and colleagues find differences after immunization of mice with 3 BCG sub-strains in the number of IFNγ-producing splenic and lung T cells [15]. However, this did not translate to differences in protection from an in vivo infection. Interestingly, the immune profile changes over time, but these changes were not translated to in vivo protection [16]. This is in accordance with our data, as no difference in lung or spleen bacterial burden was found after *Mtb* challenge in mice immunized with BCG Pasteur Aeras or BCG SSI (Fig. 3). The MGIA using BCG SSI was carried out 4 weeks after immunization, whilst the assay using BCG Pasteur Aeras was carried out 6 weeks after immunization (Fig. 1). It is possible that a difference in immune responses at different time points would be apparent in an *ex vivo* MGIA, which might contribute towards the differences seen in Fig. 1, but not after challenge in vivo. We observed a faster growth rate in vitro of BCG Pasteur Aeras compared to BCG SSI (data not shown). This could be an additional reason for the larger differences between control and immunized groups seen with this strain *ex vivo*, if faster growth of bacteria is found in the control groups with comparable growth inhibition in the immunized groups. The immune response to BCG immunization was not characterized here, and it is therefore difficult to conclude which factor is the main driver behind the observed differences. The MGIA at this stage is not meant to exactly reproduce in vivo effects, but is a step that provides additional information between assays determining immunogenicity and in vivo challenge models. As a better indication of immunization-mediated growth inhibition was seen using the faster growing BCG Pasteur Aeras, this strain was used for subsequent experiments.

We reasoned that vaccine-mediated growth inhibition would depend on the presence of mycobacterial antigen-specific T cells. As total splenocytes are used in this assay, the proportion of antigen specific T cells in the total population is not known, and the total number may be too low to have an effect on growth inhibition when using low numbers of splenocytes. Similarly, the ratio of monocytes to mycobacteria may influence the outcome as mycobacteria can survive in these cells. In order to maximize growth inhibition in immunized compared to control splenocytes, we investigated several combinations of total host cell numbers (1, 3, or 5 × 10^6^) and inocula of BCG Pasteur Aeras (2000, 500, or 100 CFU; Fig. 2). This confirmed that a lower number of bacteria leads to a greater difference between the groups, regardless of the number of host cells. We also observed that a higher number of host cells increased the difference between groups. Overall, we determined that 5 × 10^6^ splenocytes and 100 CFU result in a comparatively large and statistically significant difference between medians of 0.81 log10 CFU whilst variability is acceptable (Coefficient of Variation = 22.63 % in the BCG group; 8.06 % in the control group; Fig. 2d). A significant difference was detectable in three out of four independent experiments, indicating that some variability remains and that controls of BCG immunized and naïve animals should be included when testing experimental TB vaccine candidates (Additional file 2: Figure S2). Analysis of variance of the data shown in Fig. 2a-c suggests that sample sizes of 6–8 are required to achieve statistical power to detect an effect size of >0.5 log10 CFU. Therefore increasing group sizes from the numbers used here (5–6 per group) may increase overall reproducibility.

Using fast growing mycobacteria such as *M. smegmatis* also led to growth inhibition *ex vivo* after immunization with BCG Pasteur Aeras (Fig. 4). The effect size seen here was similar to the one found when using BCG Pasteur Aeras as the target bacteria. We argued that the faster growth rate of Msm would lead to a shorter study time, that the non-virulent nature of the bacteria would ease handling and allow this assay to be carried out in a wider range of laboratories, and that a faster growth rate may lead to an accentuation of the effect size of growth inhibition. The overall experiment time was reduced by approx. 7 days. However, we did not find more pronounced growth inhibition. This may be because the non-pathogenic nature of the bacteria does not allow it to grow proportionately faster intracellularly in cells from non-immunized mice, or there may not be enough shared antigens between the BCG strain used for immunization and the target of growth inhibition (*Msm*).

Importantly, both assays result in inhibition of mycobacterial growth by up to 0.7–0.8 log10 CFU. This is within the range of the reduction of bacterial burden seen in our own challenge with *Mtb* Erdman, where we found a reduction of approx. 0.7 log10 CFU in lungs and 1 log10 CFU in spleens of immunized animals (Fig. 3). Others have found varying degrees of reduction in bacterial burden depending on the *Mtb* challenge strain used (0.75–1.26 log10 CFU in lung, 0.48–1.32 log10 CFU in spleen) [17].

We did not carry out any assessment of *ex vivo* growth inhibition using *Mtb* as the target bacteria, and it is therefore uncertain how representative these conditions are of an infection with virulent *Mtb*. However, using a growth inhibition assay that involves co-culture of mycobacteria-infected bone marrow-derived macrophages with non-adherent splenocytes from mice vaccinated with different vaccines, Kolibab and colleagues show that in vitro growth inhibition of BCG correlates with in vitro growth inhibition of *Mtb*, as well as with in vivo challenge with *Mtb* [18]. We are presenting here a proof-of-concept study to enable further development of this assay. The advantage of using BCG as the target bacteria is that no specialized containment facilities are needed, making the MGIA much more accessible for a wide range of laboratories.

Using splenocytes from IFNγ^-/-^ mice, we examined whether the MGIA can be used to assess the impact of immune factors such as IFNγ-dependent activity on growth control of mycobacteria. We found that splenocytes from IFNγ^-/-^ mice are more permissive to mycobacterial growth than their WT counterparts (Fig. 5). This is in accordance with a wide array of literature describing the crucial role of IFNγ during tuberculosis [12, 13, 19, 20].

The main factor driving the outcome of the MGIA is currently unclear. However, given that the *ex vivo* MGIA directly assesses the summative ability of the host immune system to inhibit mycobacterial growth, the vaccine-mediated immune mechanism that underlies growth control does not need to be known a priori. In fact, this assay could help to determine underlying immune mechanisms of protection by investigating common factors in samples with efficient growth inhibition. As an *ex vivo* assay, it is also easily manipulated, for example by adding or depleting sub-populations of cells or cytokines. A further strength lies in the fact that several *Mtb* strains could be tested by using one single group of immunized mice and incubating aliquots of splenocytes with the different *Mtb* strains. This would significantly reduce the number of animals needed in comparison to in vivo challenge experiments, which need one group of animals for each *Mtb *strain to be tested – an ethical consideration that is regarded a priority within the UK research community. This approach may further allow the comparison of immune mechanisms elicited by the different strains.

## Conclusions

We have taken several steps to optimize an existing mycobacterial growth inhibition assay in order to provide a potential tool that could help accelerate TB vaccine development. We titrated both host splenocyte numbers and bacterial inocula, and found three conditions that fulfill our criteria of delta >0.5 log CFU, Coefficient of Variance < 50 %, and required sample size < 10 per group (Fig. 2d). Whilst this is an improvement over the previously published 0.2 log10 CFU difference [11], variability and reproducibility could be further optimized. However, this assay could be useful in pre-clinical testing of vaccine candidates, as an intermediate step between immunogenicity testing and in vivo challenge experiments. It is both more rapid and economic than in vivo challenge experiments, and would also significantly reduce the potential harm experienced by the animals during such experiments. With an MGIA vaccine candidates could be screened in multiple doses, with different adjuvant formulations or against different lineages of *Mtb* before proceeding to in vivo efficacy testing. One of the strengths of this assay is its potential to be translated to other species, and therefore the possibility to test the same vaccine candidate in several animal models without the need for a pathogenic challenge experiment, or in humans.

The *ex vivo* MGIA is an important tool for the TB vaccine community and we encourage others to include this assay into their studies, and to contribute to the characterization and optimization of parameters will drive the development of the MGIA forward.

## Abbreviations

BCG, Bacillus Calmette-Guérin; CFU, colony forming unit; IFNγ, interferon gamma; MGIA, mycobacterial growth inhibition assay; Msm, Mycobacterium smegmatis; Mtb, Mycobacterium tuberculosis; PBS, phosphate buffered saline; TB, tuberculosis; TTP, time to positivity; WT, wild type

## Additional files

Additional file 1: Figure S1. Standard curve of BCG Pasteur Aeras used to convert TTP to CFU. A linear regression analysis was carried out in GraphPad Prism. The resulting equation was used to calculate log10 CFU. (PDF 62 kb) Additional file 2: Figure S2. Reproducibility of *ex vivo* MGIA. A) 5 × 10^6^ splenocytes from mice immunized with BCG Pasteur Aeras (grey circles) or given saline (open squares) were co-cultured with 100 CFU of BCG Pasteur Aeras in four separate experiments. The MGIA was carried out 6 weeks after immunization with the exception of Exp. 2 (5 weeks after immunization). B) Data pooled from A). Each data point represents one animal. Error bars represent the median +/- interquartile range. Statistical significance was tested using the unpaired t test function in GraphPad Prism. (PDF 105 kb)
