# Supplementary figures and images for: A new tool for tuberculosis vaccine screening: Ex vivo Mycobacterial Growth Inhibition Assay indicates BCG-mediated protection in a murine model of tuberculosis
Source: BMC Infect Dis. 2016 Aug 12;16:412. doi: 10.1186/s12879-016-1751-4 (PMC4983071; doi:10.1186/s12879-016-1751-4)

# BCG Pasteur Aeras MGIT standard curve

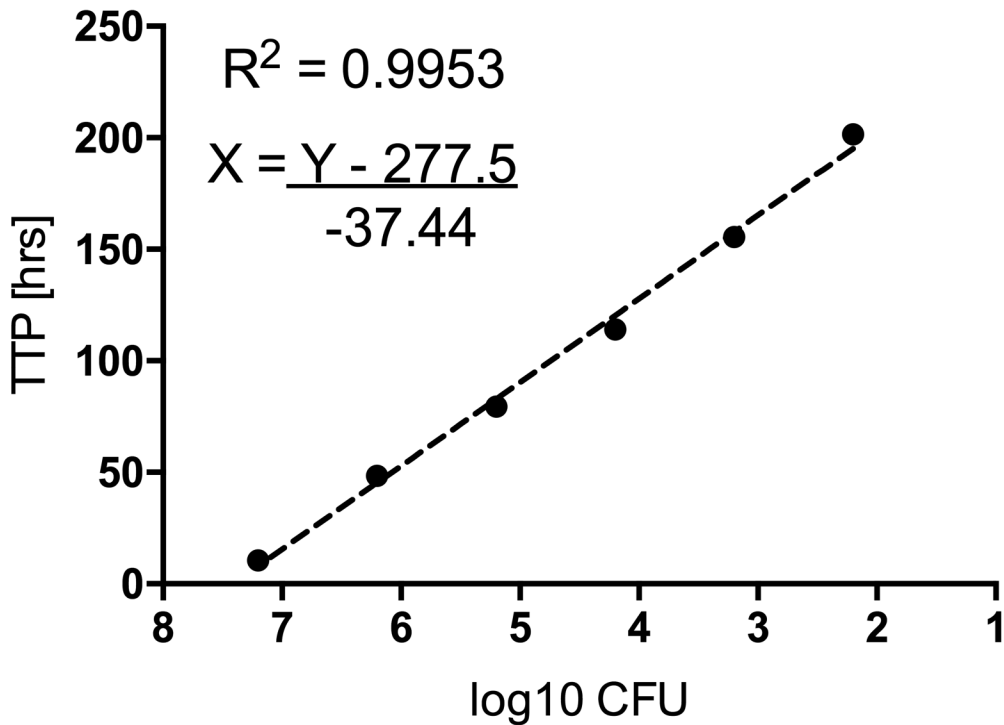

Supplement: Additional file 1: Figure S1. — Standard curve of BCG Pasteur Aeras used to convert TTP to CFU. A linear regression analysis was carried out in GraphPad Prism. The resulting equation was used to calculate log10 CFU. (PDF 62 kb) [file 12879_2016_1751_MOESM1_ESM.pdf]

**A**total log<sub>10</sub> CFU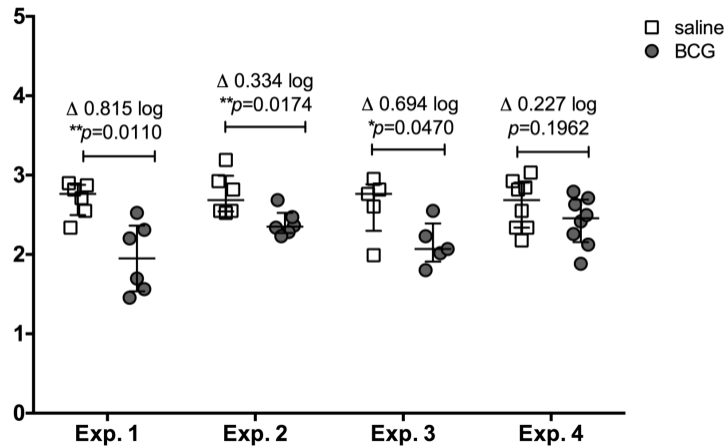**B**total log<sub>10</sub> CFU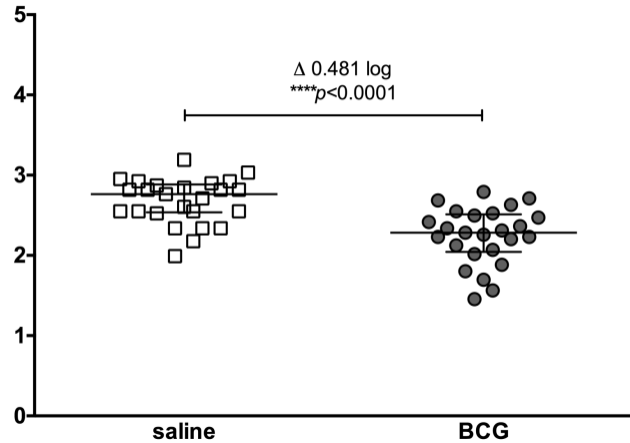

Supplement: Additional file 2: Figure S2. — Reproducibility of ex vivo MGIA. A) 5 × 10^6^ splenocytes from mice immunized with BCG Pasteur Aeras (grey circles) or given saline (open squares) were co-cultured with 100 CFU of BCG Pasteur Aeras in four separate experiments. The MGIA was carried out 6 weeks after immunization with the exception of Exp. 2 (5 weeks after immunization). B) Data pooled from A). Each data point represents one animal. Error bars represent the median +/- interquartile range. Statistical significance was tested using the unpaired t test function in GraphPad Prism. (PDF 105 kb) [file 12879_2016_1751_MOESM2_ESM.pdf]
